# Surgery of Stomach

**Published:** 1904-12-31

**Authors:** 


### Progress in Medicine and Surgery.

### SURGERY OF STOMACH.

Gastric Ulcer, according to Mayo,[1] has an obscure etiology. The three known factors of great importance are hyperchlorhydria, anæmia, and mechanical injury. Chronic ulcer of the duodenum should be classed with gastric ulcer. It is essentially a disease of adult males. The symptoms of chronic ulcer are variable, but the chief one is pain. Indications for the surgical treatment of gastric ulcer can be divided into two distinct classes: First, the ulcer itself; second, the complicating adhesions, deformities, obstructions, and so forth. There are clinically three varieties of gastric ulcer: the mucous erosion, limited to the superficial epithelium; the round and fissure ulcer, limited to the mucous coat, except in perforative cases; the large irregular ulcer, invading all the coats of the stomach. The keynote to the surgery of gastric ulcer is drainage, and the best place for this is to the left of the muscular pyloric part. He speaks highly of Finney's method of gastro-duodenostomy. Murdoch[2] says gastric ulcers are multiple in 54 per cent.; hæmorrhage occurs as a first symptom in 75 per cent.; and perforation as a first symptom in 3 per cent. Chronic gastric ulcers rarely occur before the age of 30, and are solitary in 87 per cent. Immediately fatal hæmorrhage occurs in from 3 to 5 per cent., and perforation in 7 per cent. Hæmorrhage occurs at some time in 18 per cent. of cases. The indications for operation are perforation and hæmorrhage, although, recently, well-known surgeons have advocated operation for the cure of ulcer itself. The death-rate, however, from chronic gastric ulcer under proper medical treatment is not more than 4 per cent., whereas the mortality following gastro-enterostomy for benign stenosis of the pylorus is from 10 to 50 per cent. An operation is advisable if the ulcer is bleeding and if this condition lasts for weeks. Patients with chronic gastric ulcer should be kept on liquid food, not for weeks, but for months, or until the ulcer heals. Recurrence happens in about 6 per cent. of cases. Murdoch,[3] quoting Fenwick, says the principal forms of chronic ulcer are as follows:—(1) The gastralgic form, characterised by attacks of severe pain, resembling biliary colic. (2) The catarrhal or vomiting form, which may be confounded with acute gastric catarrh, hysteria, or uncontrollable vomiting of pregnancy. (3) The dyspeptic form, which may be confounded with cirrhosis of the liver and thoracic aneurysm, in both of which diseases hæmatemesis may occur. (5) The cachectic form, characterised by emaciation, debility, and cachexia, so that it may be difficult to distinguish it from cancer or pernicious anæmia. Under the most favourable circumstances a chronic ulcer requires many months for its cure, and the mere subsidence of pain and vomiting is no proof that cicatrisation has taken place. He divides the period of treatment into four stages. The first period lasts for two or three weeks, during which the patient remains in bed and has of course only liquid food. The second period lasts from the end of the second or third week to the end of the second month, during which time rest is advised and the liquid food is continued, with the addition to the milk of ground rice, flour, powdered biscuit, or tapioca, clear soups, and expressed meat-juice. The third period extends from the beginning of the third to the end of the sixth month. Although milk continues to be the staple article of diet, the patient may be permitted to have bread and milk, bread-and-butter, poached eggs, scraped and pounded raw meats, chicken cream, and broiled white fish which has passed through a sieve. The fourth and final period should extend from the sixth to the twelfth or eighteenth month, according to the severity of the case. During this time the diet is gradually increased until at its termination there are only a few articles which have to be prohibited. He does not advocate gastro-enterostomy for the cure of the ulcer, but only for its sequelæ, such as pyloric obstruction or atony of the gastric muscle. Turner and English[4] give nine consecutive cases of perforated gastric ulcer treated by suture, irrigation, and drainage.

Gastro-enterostomy has been performed for a variety of purposes which Barling[5] classifies into three groups: (1) For stenosis of the pylorus, for hour-glass contraction of the stomach, or for stenosis of the duodenum, in each case to allow the contents of the stomach to pass freely into the intestinal tract, and thus provide relief to gastric stasis, with its distressing symptoms and the incapacity for work which it brings. (2) For the purpose of putting the stomach at rest, to stop hæmorrhage from ulcers, whether situated in stomach or duodenum, with the intention also of relieving pain and procuring the healing of the ulcers. (3) For atonic dilatation of the stomach with marked proptosis, it being hoped that the substitution of a passive opening for the pylorus, and at a more dependent position of the stomach, would relieve the organ of its burden of over-fulness and consequent pain and vomiting. Chronic dyspepsia, without evidence of gastric ulcer or dilatation, has also thus been treated as a refuge when no relief was obtained by dieting or other medical measures. The last group is the most unsatisfactory. Taking the whole of the cases together, pyloric stenosis has been the reason for surgical interference in a large majority. Dr. Saundby thinks that the minor degrees of obstructive dilatation are often overlooked, as in them the classical symptoms, periodic vomiting and visible peristalsis, are absent, and the symptoms are ascribed to chronic gastritis. He holds that the only way to avoid this mistake is by the systematic employment of inflation in the examination of stomach cases. But dilatation of the stomach is not always dependent upon pyloric obstruction, and where it is due simply to atony experience shows that surgical interference does not give good results. It is therefore important to determine whether obstruction exists or not. Dr. Saundby believes that this may be done by the examination of the fasting stomach twelve hours after the taking of a test meal of bread and meat the night previously. If there is no obstruction the stomach will be completely free from even microscopical traces of food. If there is slight obstruction there may be traces of food, the amount of residue being in proportion to the degree of obstruction. Barling has found this method useful, but thinks it has been too recently introduced to enable a final opinion to be given as to its value. Rogers[6] says two causes, which are to a considerable extent capable of elimination, seem to be visible in nearly all of the bad results, and modifications of the original operation have been brought forward to correct them. One of these causes has to do with the ever-present but unforeseeable possibility of occlusion of the alimentary tract at or near the point of anastomosis, and the other, which follows the first almost as a corollary, is dependent upon the fact that patients often do not submit to operation until they have reached a serious condition of exhaustion. The cause of death as described in their usual order of frequency are shock, collapse, asthenia, or inanition, all practically the same when one bears in mind the extreme debility of the patients usually considered to require so (statistically) serious an interference. Roux's method "en Y" attempts to solve the difficulty of the loop by section of the gut at the summit with implantation of the distal end into the stomach, and of the proximal end into the intestine below; but the numerous unpleasant possibilities, the chief of which are the time it requires and the increased risk of infection, have not made it popular. Petersen's operation does not involve the use of any loop, and he reports 215 gastro-enterostomies, with only a 5 per cent. mortality from all causes in benign cases, and among these there was not a single instance of serious vomiting, and no obstruction. It is based on the simple anatomical principle that the first few inches of the jejunum lie directly in contact with the posterior surface of the stomach, separated from it only by the non-vascular portion of the mesocolon, and that this part of the jejunum descends vertically and without any turns. It is attached two to four inches below its origin, lying vertically, and is thus anastomosed in its normal position, and without any twists, to the adjoining posterior stomach wall. Deaver[7] says that his experience has taught him to perform the posterior method of anastomosis, or the Roux Y operation, and, in the event of vomiting following the former method, he would re-operate, divide the afferent limb just below the stomach, and implant it, as in Roux Y, into the efferent limb some four or six inches below the stomach, closing the shallow sac above close to the anastomotic opening. The after-treatment of a patient upon whom gastro-enterostomy has been performed is of great importance. Shock, vomiting, and pneumonia should be guarded against at the time of operation by the use of a hot-water bed, hot bottles, and a cotton jacket. If shock occurs saline solution by the bowel is very useful, at first combined with whisky, and later intravenously, if the condition of the patient should demand it. Vomiting is greatly to be feared, but he allows liquid food 18 hours after operation unless there is nausea. If vomiting does occur the best treatment is gastric lavage, using small quantities of water at a time—200 or 300 c.c.—and giving absolutely nothing by the mouth. Should peritonitis set in a re-operation is imperatively demanded, the anastomosis strengthened, and the peritoneum washed out. If the shock does not prove fatal, such a patient should recover, as the infection from the fasting stomach is not of a very virulent type. Robson[8] states that the operation of gastro-enterostomy is imperative in the following conditions as soon as the diagnosis is established:—(1) In cases of pyloric stenosis leading to dilatation of the stomach. (2) In malignant stenosis of the pylorus. (3) A degree of congenital stenosis of the pylorus which causes, though often unrecognised, a dilatation of the stomach in young adults. (4) Congenital atresia of the pylorus, in which no passage exists between the stomach and intestine. (5) In chronic ulcer, with tumour of doubtful character, too extensive or adherent for effectual removal. (6) In cancer or tumour of the duodenum, producing obstruction to the onward passage of the gastric contents. (7) Hour-glass contraction of the stomach due to chronic ulcer or to cancer; but great care must be observed that the junction is not made between the bowel and the distal cavity, otherwise no benefit will ensue. (8) In perigastric adhesions. (9) In tumour outside the pylorus, but pressing on it and causing obstruction to the onward passage of the stomach contents. (10) In ulcer of the stomach, whether acute or chronic, not yielding to medical treatment. (11) Duodenal ulcer, which fails to yield to general treatment. (12) In hæmorrhage from the stomach or duodenum, where general treatment has been tried and failed, and where the bleeding is persisting or recurring after brief intervals. (13) In persistent spasm of the pylorus, or Reichmann's disease, leading to dilatation of the stomach. (14) Hyperchlorhydria, which, in spite of treatment, causes much misery by constant indigestion with acid eructations. (15) Persistent gastralgia. (16) In tetany of gastric origin. Death occurs in 75 per cent. of the cases of tetany occurring in gastric dilatation. (17) Acute gastric dilatation. (18) In an exceptional case of ulcer of the stomach eroding the pancreas and producing pancreatitis, with abscess draining into the stomach. (19) Cholelithiasis, leading to such extensive perigastritis and thickening of the pylorus that gastrolysis is not sufficient. (20) In some cases of atonic dilatation of the stomach. Moynihan[9] records 85 cases of gastro enterostomy for chronic ulcer with intractable dyspepsia, or dilated stomach, with one death; and 15 cases for profuse and recurring hæmorrhage with one death. The correct explanation of regurgitant vomiting was first given by Mayo. He showed that if the anastomosis were made at some distance from the greater curvature on either the anterior or the posterior surface, a pool of fluid collected below the opening and then, being stagnant, excited the act of vomiting. Moyniham says he has never seen the vomiting of bile since making sure that the lower end of the opening was at the greater curvature. There are cases, however, to which this explanation does not apply. In these there is an acute kink at the point of anastomosis, and the symptoms are those of intestinal obstruction high in the small intestine. In such circumstances the bile and pancreatic juice regurgitate into the stomach through the pylorus.

[1] Med. Rec., April 23, 1904, p. 668. [2] Ibid., May 28, 1904, p. 902. [3] Ibid., Oct. 1, 1904, p. 529. [4] Lancet, July 16, 1904, p. 145. [5] Brit. Med. Jour., May 7, 1904, p. 1064. [6] Annals of Surg., April 1904, p. 512. [7] Amer. Jour. Med. Sci., Feb. 1904, p. 187. [8] Ibid., p. 247. [9] Annals of Surg., May 1904, p. 690.

*(To be continued.)*

